# Racial disparities in end-stage renal disease in a high-risk population: the Southern Community Cohort Study

**DOI:** 10.1186/s12882-019-1502-z

**Published:** 2019-08-07

**Authors:** Fabian Bock, Thomas G. Stewart, Cassianne Robinson-Cohen, Jennifer Morse, Edmond K. Kabagambe, Kerri L. Cavanaugh, Kelly A. Birdwell, Adriana M. Hung, Khaled Abdel-Kader, Edward D. Siew, Elvis A. Akwo, William J. Blot, T. Alp Ikizler, Loren Lipworth

**Affiliations:** 1 Division of Nephrology and Hypertension, Department of Medicine, Vanderbilt University Medical Center, Nashville, TN USA; 2 Department of Biostatistics, Vanderbilt University Medical Center, Nashville, TN USA; 3 Division of Epidemiology, Department of Medicine, Vanderbilt University Medical Center, 2525 West End Ave, Ste 600, Nashville, TN 37203 USA; 4 Vanderbilt-O’Brien Center for Kidney Disease, Vanderbilt University Medical Center, Nashville, TN USA

**Keywords:** End-stage renal disease, Disparity, Race, Case-cohort study, Cardiovascular risk factors, Chronic kidney disease, Socioeconomic status

## Abstract

**Introduction:**

The Southern Community Cohort Study is a prospective study of low socioeconomic status (SES) blacks and whites from the southeastern US, where the burden of end-stage renal disease (ESRD) and its risk factors are high. We tested whether the 2.4-fold elevated risk of ESRD we previously observed in blacks compared to whites was explained by differences in baseline kidney function.

**Methods:**

We conducted a case-cohort study of incident ESRD cases (*n* = 737) with stored blood and a probability sampled subcohort (*n* = 4238) and calculated estimated glomerular filtration rate (eGFR) from serum creatinine. 86% of participants were enrolled from community health centers in medically underserved areas and 14% from the general population in 12 states in the southeastern United States. Incident ESRD after entry into the cohort was ascertained by linkage of the cohort with the US Renal Data System (USRDS).

**Results:**

Median (25th, 75th percentile) eGFR at baseline was 63.3 (36.0, 98.2) ml/min/1.73m^2^ for ESRD cases and 103.2 (86.0, 117.9) for subcohort. Black ESRD cases had higher median (25th, 75th) eGFR [63.3 (35.9, 95.9)] compared to whites [59.1 (39.4, 99.2)]. In multivariable Cox models accounting for sampling weights, baseline eGFR was a strong predictor of ESRD risk, and an interaction with race was detected (*P* = 0.029). The higher ESRD risk among blacks relative to whites persisted (hazard ratio: 2.58; 95% confidence interval: 1.65, 4.03) after adjustment for eGFR.

**Conclusion:**

In this predominantly lower SES cohort, the racial disparity in ESRD risk is not explained by differences in baseline kidney function.

**Electronic supplementary material:**

The online version of this article (10.1186/s12882-019-1502-z) contains supplementary material, which is available to authorized users.

## Background

Chronic kidney disease (CKD) is one of the fastest growing chronic health conditions worldwide, particularly among minority populations, and is associated with substantially increased risks of end-stage renal disease (ESRD) and cardiovascular mortality [1, 2]. In 2015, there were 124,114 incident cases of ESRD in the United States. ESRD incidence rates for blacks are more than three times greater than for whites, at 865 per million in 2013 [3]. There is a striking disparity in the lifetime risk of developing ESRD: 8% among blacks compared to 2–3% among whites [4]. To identify specific subpopulations at risk and their risk factors, a number of cohort studies have examined racial differences in ESRD and have consistently demonstrated higher ESRD incidence among blacks compared to whites [5–7]. Neither traditional risk factors including diabetes and hypertension, nor the presence of high risk genotypes, fully explain the racial differences in ESRD [5–8], emphasizing the need for longitudinal cohort studies in vulnerable populations to examine novel factors underlying racial differences.

The Southern Community Cohort Study (SCCS) is a large ongoing, prospective study of black and white participants residing in the southeastern United States, where rates of ESRD are among the highest in the nation due to reasons that remain to be fully elucidated [3]. The age-adjusted ESRD incidence rate in the SCCS study population is 79/100,000 person-years among whites compared to 285/100,000 person-years among blacks. The SCCS provides unique advantages for examining racial differences in ESRD, since all participants have similar (typically low) household income and education levels regardless of race, limiting confounding by socioeconomic differences. In an earlier report we described ESRD incidence among black and white participants in the SCCS and its association with traditional risk factors such as male gender, low income, diabetes, and hypertension [9]. While the pattern of risk factors was similar for blacks and whites, stronger associations were observed for traditional ESRD risk factors in blacks. After adjustment for these known risk factors, the risk of ESRD nonetheless remained more than two-fold higher for blacks [hazard ratio (HR): 2.4, 95% confidence interval (CI): 1.9, 3.0]. However, these analyses lacked data on baseline kidney function. We hypothesized that the observed racial disparity in risk may be explained by differences in kidney function at baseline. To address this, we used a case-cohort design, measured baseline serum creatinine to characterize kidney function, and evaluated associations with ESRD risk among black and white participants of the SCCS.

## Methods

### Study population and data collection

The SCCS enrolled nearly 86,000 adults, age 40–79 years, residing in 12 states in the southeastern United States during 2002–2009. Approximately 86% of participants were enrolled at participating community health centers (CHC), institutions which provide primary health and preventive services in medically underserved areas [10]. A detailed description of SCCS methods is available on the study website (http://www.southerncommunitystudy.org/) and in previous publications [9, 11–13]. SCCS participants provided written informed consent, and protocols were approved by the Institutional Review Boards of Vanderbilt University Medical Center and Meharry Medical College.

Analyses were restricted to black and white individuals enrolled at CHCs, in order to ensure similar socioeconomic status and generally equal access to health care regardless of race, and the opportunity to donate a blood specimen at baseline. The SCCS does not have sufficient sample size for stable statistical analyses in other race groups. Upon enrollment at the CHC, participants were administered a baseline computer-assisted personal interview. The questionnaire ascertained information about demographic, socioeconomic and lifestyle characteristics, personal and family medical history, height, weight, and other factors (available at www.southerncommunitystudy.org).

### Blood sampling and eGFR determination

Approximately 46% of the cohort donated a baseline 20 mL blood sample during their CHC recruitment. Samples were refrigerated immediately after collection and then shipped cold on that day. Blood was processed and stored frozen at − 80 °C in the SCCS Biospecimen Repository at Vanderbilt University Medical Center on average within 1.2 days (range 1–5) after collection. Baseline serum levels of creatinine were measured using the modified Jaffe (Rate) method on a Beckman Coulter DXC 600 clinical chemistry analyzer. The creatinine assays were calibrated and daily quality checks performed at three levels before sample testing. Creatinine data were used for estimation of baseline glomerular filtration rate (eGFR) using the CKD-EPI equation [14].

### ESRD assessment

**Fig. 1:**
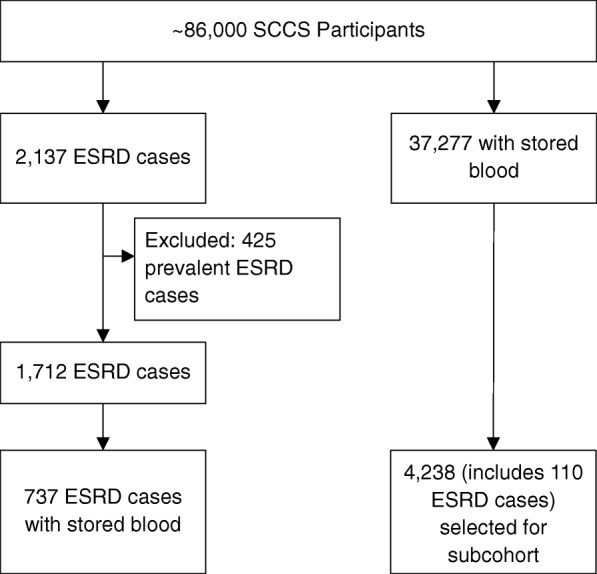
Flow chart of case-cohort design, Southern Community Cohort Study 2002–2009

Incident diagnoses of ESRD among SCCS participants after entry into the cohort were ascertained by linkage of the cohort, using date of birth, Social Security number, and first and last name, with the US Renal Data System (USRDS) from January 1, 2002 to March 31, 2015, the latest date for which data were available. The USRDS registers ESRD cases certified by a physician diagnosis and filed using a medical evidence report form (to the Medicare ESRD program) or when there is other evidence of chronic dialysis or a kidney transplant. Through March 31, 2015, a total of 2137 cases of ESRD were identified in the SCCS. We excluded from our analysis 425 individuals with a diagnosis of ESRD recorded in the USRDS prior to enrollment in the SCCS (prevalent cases). After these exclusions, there were 1712 incident cases of ESRD among SCCS participants, 737 of whom had a stored serum sample allowing for measurement of baseline creatinine (Fig. 1).

### Study design and statistical analyses

We used a case-cohort study design [15], selecting all cases of incident ESRD who had stored serum and a probability sample of the entire cohort who donated blood samples (i.e., the subcohort). The subcohort was constructed from several previous nested case-control studies performed within the SCCS for which creatinine had already been measured. Because the matching algorithm was known for each of the nested case-control studies, we were able to calculate the sampling probability into the subcohort from the entire SCCS population with donated blood. We were able to verify that all but 0.5% (209/37,277) of the SCCS population with donated blood had a positive probability of being selected into the subcohort. Using the sampling probabilities, we constructed trimmed inverse sampling probability weights. Participants were considered at risk from the date of entry into the SCCS cohort until the first occurrence of incident ESRD diagnosis, date of death, or end of available follow-up, set to the end of USRDS data availability (March 31, 2015).

We selected a weighted probability sample of 4238 participants from the 37,277 cohort participants with available blood specimens as outlined above (Fig. 1). This weighted probability sample constitutes 13% of all SCCS participants who donated blood samples, and is comparable to those participants with respect to baseline demographics and other characteristics, including racial distribution, low income and education level and high prevalence of cardiovascular risk factors (Table 1). In particular, the weighted subcohort included 70.8% blacks and 29.2% whites, which was similar to 67.3% blacks and 28.6% whites in the SCCS target population. In the overall SCCS with stored blood as well as the subcohort, about 32% had an education level < 12th grade, 60–63% had an annual income <$15,000, and approximately 55 and 22% had hypertension and diabetes, respectively.

**Table 1:** Baseline characteristics of ESRD cases, weighted subcohort, and overall SCCS population who donated blood at enrollment, 2002–2009

Characteristic	ESRD Cases *N* = 737	Subcohort *N* = 4238	SCCS (with stored blood) *N* = 37,277
Age at enrollment, median (25th, 75th percentile), years	53 (47, 59)	50 (45, 58)	50 (45, 57)
eGFR, median (25th, 75th percentile), ml/min/1.73m^2^	63.3 (36.0, 98.2)	103.2 (86.0, 117.9)	
eGFR, %, ml/min/1.73m^2^			
≤ 30	19.9	1.0	
31–60	26.0	4.8	
61–90	23.5	24.6	
> 90	30.6	69.6	
Female, %	52.6	58.8	59.5
Race-Sex categories, %			
Black women	43.6	40.0	40.0
Black men	43.4	30.8	30.1
White women	9.0	18.8	19.5
White men	4.0	10.4	10.3
Marital status, %			
Married	32.8	31.1	32.4
Separated	31.6	35.1	35.4
Widowed	12.2	10.7	9.6
Single	23.4	23.1	22.6
Education <12th grade, %	40.7	32.8	32.4
Income < $15,000, %	66.6	62.5	60.7
BMI, median (25th, 75th percentile), kg/m^2^	31.2 (26.3, 37.8)	29.2 (24.8, 34.3)	29.3 (25.1, 34.9)
BMI categories, %			
Underweight (< 18.5)	0.5	1.6	1.1
Normal (18.5–24.9)	17.8	24.2	23.4
Overweight (25–29.9)	27.7	28.7	29.1
Obese (30+)	54.0	45.6	46.5
Smoking status, %			
Current	34.5	47.1	44.6
Former	24.0	20.3	21.3
Never	41.5	32.5	34.1
Hypertension, %	85.9	54.9	56.2
Diabetes, %	68.7	22.4	22.2
Stroke/TIA, %	12.6	6.9	6.6
MI/Bypass, %	14.5	7.0	7.1

*Abbreviations*: *BMI* body mass index, *eGFR* estimated glomerular filtration rate, *MI* myocardial infarction, *SCCS* Southern Community Cohort Study, *TIA* transient ischemic attack

Using methods appropriate for probability samples (or survey samples), we described characteristics of subjects in the case-cohort stratified by race and baseline eGFR. Specifically, variable means, standard deviations, or categorical proportions were calculated using the sample weights. We modeled time to ESRD as a function of race, demographic (age at enrollment, sex), socioeconomic (education <12th grade, income < $15,000/year), and clinical variables, including baseline eGFR, body mass index (BMI, kg/m^2^), history of smoking (ever, never), and history of diabetes (yes/no and duration of diabetes), stroke, myocardial infarction, and hypertension (all yes/no), in a series of Cox regression models that accounted for the case-cohort study design and the weighted sample [16]. To allow for the potential non-linear associations between the continuous predictors (age, eGFR, and BMI) and time to ESRD, these predictors were added to the model as restricted cubic splines with four knots. To further adjust for potential confounding by diabetes severity and control, we added medication use for diabetes (including insulin to the models). In order to understand the impact of adjustment for eGFR on the association of race with ESRD and whether there is an interaction between race and eGFR on the ESRD risk, two additional models were constructed: one without eGFR altogether and another with an interaction term between race and eGFR. We used the complete case method; 217 (4.56%) participants were missing one or more covariates and excluded from the Cox proportional hazards analyses.

From the Cox models, we calculated hazard ratios and corresponding 95% confidence intervals. In addition to hazard ratios, we constructed partial effect plots of eGFR and race on the log relative hazard scale, which highlight the differences in ESRD risk between blacks and whites for different levels of baseline kidney function while adjusting for the other variables in the model. The partial effect plot in this analysis compared the log relative hazard of ESRD as a function of eGFR while holding all other covariates to the same value. In short, the partial effect plot is generated by calculating the log relative hazard from the Cox model by plugging in reference values for all covariates in the regression model. For example, age was set to 55 and sex was set to female. The covariate on the x-axis, eGFR, was set to the range of values observed in the study population. Thus, by holding the other covariates constant, we can trace out the shape of the associate between eGFR, race, and the log relative hazard of ESRD.

**Fig. 2:**
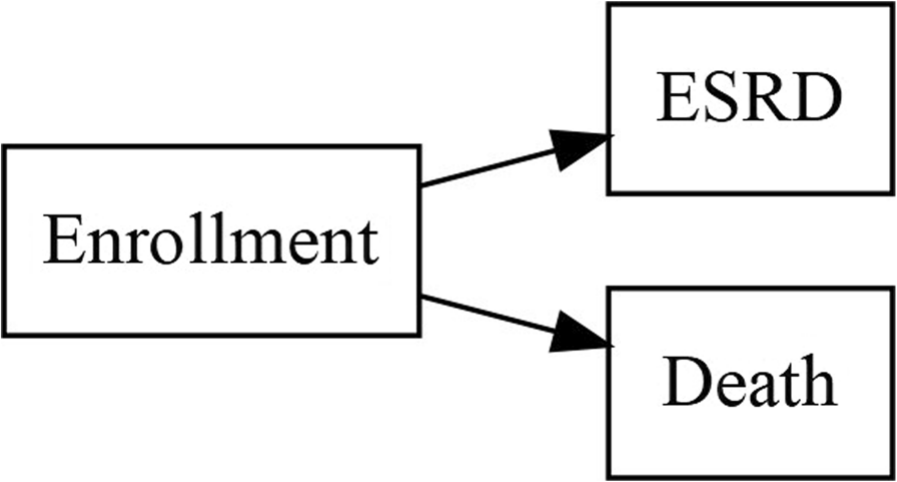
Multistate Model

The Cox proportional hazards model for time to ESRD is often referred to as a cause-specific analysis because death is treated as a censoring event. Cause-specific analyses are routinely performed, and the resulting HR are interpreted similarly to standard HR (under the assumption that the time-to-event distribution is independent of the censoring distribution which includes death). Estimating the cumulative incidence function from a cause-specific model, however, is generally not appropriate. To tease out the impact of death as a competing risk for ESRD, we constructed a multistate model (Fig. 2).

The model is time-to-first event, as death after ESRD was not modeled. The extra sub-cohort cases that enriched the case-cohort analysis with additional events were omitted from the multistate analysis. The Aalen-Johansen estimate of the probability of being in each state was generated from the cumulative hazard functions from each cause-specific model and the transition matrix implied by the model above. From the model, we generated plots of the estimated 10-year probability of incidence ESRD and death as a function of eGFR, sex which was derived from a model with age, eGFR, sex, race, diabetes status, hypertension status, and the interaction between race and eGFR. Age and eGFR were included as restricted cubic splines. All analyses were conducted using R. For main effects and interaction terms, *P* ≤ 0.05 was considered statistically significant.

## Results

Compared to the subcohort, a higher proportion of ESRD cases were black (71% vs. 87%, respectively) (Table 1). In addition, ESRD cases were slightly older at enrollment than subcohort members and were of similar socioeconomic status. In contrast to the subcohort, ESRD cases had a higher baseline prevalence of cardiovascular risk factors, notably hypertension (85.9% versus 54.9%) and diabetes (68.7% versus 22.4%). The proportions of participants with baseline eGFR ≤30, 31–60, 61–90 and ≥ 90 ml/min/1.73m^2^ in the SCCS subcohort were 1.0, 4.8, 24.6 and 69.6%, respectively (Table 1), while among those who subsequently developed ESRD they were 19.9, 26.0, 23.5 and 30.6%. Median (25th, 75th percentile) baseline eGFR among subcohort members was 103.2 (86.0, 117.9) compared to 63.3 (36.0, 98.2) among ESRD cases. The vast majority of the subcohort (94.2%) and the majority of ESRD cases (54.1%) had an eGFR > 60 at enrollment.

Black subcohort members had a higher median eGFR at baseline compared to whites (107.7 vs. 96.9 ml/min/1.73m^2^) (Table 2). Despite the higher incidence rate of ESRD in blacks, the proportions of black and white ESRD cases with normal kidney function (eGFR > 60 ml/min/m^2^) at baseline were not meaningfully different (54.7% in blacks versus 49.8% in whites). Black and white subcohort members were similar in terms of baseline characteristics, including education and income level, obesity, and the prevalence of cardiovascular risk factors (hypertension, diabetes, stroke). Among ESRD cases, blacks were younger at cohort enrollment compared to whites (median age 52 versus 59 years, respectively), and were more likely to be overweight or obese than white cases. Also, black ESRD cases were slightly more likely to have hypertension compared to white ESRD cases (86.4% versus 82.6%, respectively), but less likely to have a history of diabetes (67.8% versus 74.5%), stroke (11.3% versus 21.3%) or myocardial infarction/bypass (12.8% versus 26.2%).

**Table 2:** Baseline characteristics of ESRD cases and subcohort members according to race, SCCS 2002–2009

	Black		White	
	Subcohort	ESRD	Subcohort	ESRD
eGFR, median (25th, 75th percentile), ml/min/1.73m^2^	107.7 (88.8121.3)	63.3 (35.9, 95.9)	96.9 (82.5, 107.0)	59.1 (39.4, 99.2)
eGFR, ml/min/1.73m^2^, %				
≤ 30	1.1	20.0	0.7	19.0
31–60	4.2	25.3	6.2	31.1
61–90	21.7	25.5	31.5	10.2
> 90	73.0	29.2	61.6	39.6
Age at enrollment, median (25th, 75th percentile), years	50 (45, 56)	52 (47, 58)	53 (46, 61)	59 (54, 62)
Age categories, %				
40–49	51.7	39.6	42.6	17.9
50–59	33.7	44.1	32.3	49.1
60–69	11.6	12.5	19.0	31.7
70–79	3.0	3.8	6.2	1.3
Female, %	56.5	50.2	64.4	69
Marital Status, %				
Married	26.0	33.6	43.4	27.3
Separated	35.6	30.0	34.1	42.4
Widowed	28.0	25.6	11.4	9.1
Single	10.5	10.8	11.2	21.1
Education <12th grade, %	33.6	40.7	30.8	40.6
Income < $15,000, %	63.6	65.3	60.0	75.3
BMI, median, (25th, 75th percentile), kg/m^2^	29.4 (25.1, 34.3)	31.3 (26.6, 38.4)	28.7 (24.5, 33.9)	29.1 (23.3, 34.6)
BMI categories, %				
Underweight (< 18.5)	0.8	0.5	3.5	0.6
Normal (18.5–24.9)	23.3	15.8	26.2	31.1
Overweight (25–29.9)	29.2	28.9	27.3	19.8
Obese (30+)	46.7	54.9	43.1	48.4
Smoking status, %				
Current	47.1	32.4	47.1	48.2
Former	19.3	24.6	22.9	20.4
Never	33.6	43.0	30.0	31.4
Hypertension, %	56.3	86.4	51.4	82.6
Diabetes, %	22.4	67.8	22.2	74.5
MI/Bypass, %	5.4	12.8	10.8	26.2
Stroke/TIA, %	6.7	11.3	7.4	21.3

*Abbreviations*: *BMI* body mass index, *eGFR* estimated glomerular filtration rate, *MI* myocardial infarction, *SCCS* Southern Community Cohort Study, *TIA* transient ischemic attack

Across all baseline eGFR categories, a higher proportion of ESRD cases compared to subcohort members were black; the only apparent exception, in race-by-sex tabulations, was a lower proportion of black women among ESRD cases with eGFR > 90 compared to subcohort (33.7% vs. 45.0%) (Additional file 1: Table S1).

The median (range) time of follow up for the overall study population was 9.3 years (6.9, 11.1). In multivariable models (Table 3), after adjustment for baseline eGFR as well as clinical, lifestyle and demographic characteristics (age, sex, BMI, smoking, education, income, hypertension, diabetes, stroke/TIA and MI/bypass), the greater than two-fold elevated ESRD risk among blacks compared to whites persisted (HR: 2.58; 95% CI: 1.65, 4.03). Women were at significantly lower risk for ESRD compared to men (HR: 0.42; 95% CI: 0.31, 0.58), and diabetes duration ≥20 years was strongly associated with ESRD risk in our study population (HR: 12.17; 95% CI: 7.34, 20.16); the association between hypertension and ESRD was attenuated by adjusting for baseline eGFR but remained statistically significant (HR: 1.96; 95% CI: 1.21, 3.19). The race and sex associations with ESRD observed in the cause-specific analysis were also observed in the multi-state model (Additional file 2: Table S2 and Additional file 4: Figure S1). The addition of medication use for diabetes (including insulin) to the models did not change the results.

**Table 3:** Adjusted hazard ratios (HR) and 95% confidence intervals (CI) for the association between baseline characteristics and end-stage renal disease (ESRD), SCCS 2002–2009

	ESRD risk (unadjusted for eGFR)^a^		ESRD risk (adjusted for eGFR)^b^	
	HR	95% CI	HR	95% CI
Black race (Ref: white)	2.64	1.72, 4.04	2.58	1.65, 4.03
Female (Ref: male)	0.51	0.38, 0.70	0.42	0.31, 0.58
Education <12th grade (Ref: ≥12th grade)	1.02	0.76, 1.37	0.96	0.70, 1.31
Income < $15,000 (Ref: ≥$15,000)	1.11	0.81, 1.50	0.97	0.71, 1.33
Ever Smoker (Ref. never)	0.81	0.59, 1.09	0.70	0.51, 0.96
Hypertension	3.30	2.13, 5.11	1.96	1.21, 3.19
Diabetes Duration (Ref: no diabetes)				
< 10 years	4.04	2.75, 5.93	5.25	3.53, 7.81
10–19 years	12.57	8.44, 18.73	8.50	5.57, 12.96
≥ 20 years	16.00	10.17, 25.18	12.17	7.34, 20.16
Stroke, TIA	1.54	0.99, 2.40	1.80	1.16, 2.79
MI/Bypass	1.30	0.85, 1.99	0.98	0.63, 1.52

*Abbreviations*: *CI* confidence interval, *eGFR* estimated glomerular filtration rate, *ESRD* end-stage renal disease, *HR* hazard ratio, *MI* myocardial infarction, *SCCS* Southern Community Cohort Study, *TIA* transient ischemic attack

^a^All variables were included in the Cox regression model simultaneously, in addition to age and body mass index modeled as restricted cubic splines

^b^Additionally adjusted for eGFR at baseline

**Fig. 3:**
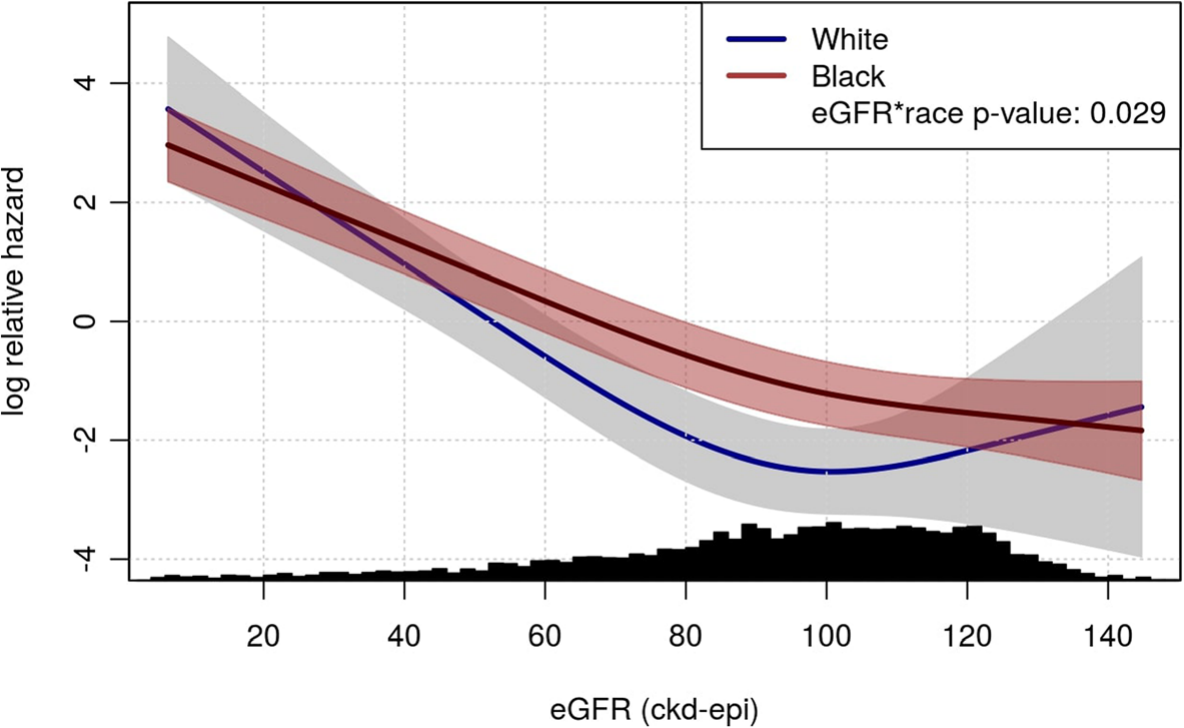
Partial effect plot of ESRD risk and baseline eGFR. Adjustment Variables: age, sex, BMI, eGFR, smoking, education, income, hypertension, diabetes, stroke/TIA and MI/bypass

In a partial effect plot based on multivariable Cox models, we examined the association between baseline eGFR and incident ESRD, by race. As shown in Fig. 3 (Additional file 3: Table S3), baseline eGFR was a strong predictor of ESRD risk. While the ESRD risk in blacks was significantly higher than the risk for whites for most levels of baseline eGFR < 100 ml/min/1.73m^2^, the curves crossed at about eGFR < 40 ml/min/1.73m^2^, demonstrating heterogeneity of the eGFR effect across racial groups (*P* for race*eGFR interaction = 0.029). Furthermore, compared to blacks, the slope of the curve for whites appeared steeper; each 10 mL/min lower eGFR at baseline was associated with a greater increase in log relative hazard of ESRD.

## Discussion

In this large case-cohort study in a population with a high burden of ESRD, we demonstrated that blacks compared to whites were at disproportionate risk for developing ESRD, after accounting for baseline kidney function and other common confounders. For most baseline eGFR values < 100 ml/min/1.73m^2^, the risk of ESRD was higher in blacks than whites. Remarkably, more than half of black participants with ESRD had an eGFR ≥60 ml/min/1.73m^2^ at baseline, with a median time from study enrollment to ESRD of 6–7 years. In addition, blacks had an age-adjusted ESRD incidence rate more than three times greater than their white counterparts despite having a marginally higher median eGFR at baseline than whites. Differences in baseline risk profiles were also apparent, with blacks who subsequently developed ESRD more likely to be obese and hypertensive while diabetes and CVD were more common among whites who developed ESRD.

The persistently higher risk of ESRD among blacks compared to whites suggests an influence of additional factors beyond those already known. Diabetes and hypertension remain the leading risk factors for ESRD. However, our study and others have previously demonstrated that these do not fully explain the racial disparity in ESRD risk [8]. In light of the relatively rapid development of ESRD described herein, the lower SES of most participants, and the availability of information on baseline kidney function, the SCCS cohort is uniquely positioned to study additional risk factors, including potentially modifiable lifestyle behaviors, for development of ESRD in this vulnerable population. The SCCS has resources available that allow further examination of metabolic and genetic profiles associated with CKD progression. Furthermore, the case-cohort design has several advantages. The subcohort we created can be used to study other outcomes in the context of chronic kidney disease and unlike in nested case-control studies the design allows for obtaining measurements at any time after the cohort was originally set up [17].

Currently, the management goals in CKD focus on influencing clinical factors (such as blood pressure and diabetes) and treating complications (anemia, acidosis, among others). However, our understanding of CKD progression is limited and effective interventions to prevent progression are lacking [18]. Apoliproprotein L1 (APOL1) risk variants, common in individuals of African ancestry, are associated with an increased risk of ESRD [19]. APOL1 affects endosomal trafficking, inflammasome activation and autophagic flux and can promote podocyte loss and glomerulosclerosis [18]. Blacks with two high-risk variants have a 7.3- to 29-fold increase in the odds of developing ESRD [20]. Therapies targeting APOL1-related injuries are currently being experimentally addressed [21]. Given its importance, clinical testing for APOL1 variants is under discussion but unexplained variance remains, and evidence of kidney disease is not observed among all carriers of two high-risk variants. Moreover, it is not known what comorbid conditions or exposures in patients with these high-risk alleles are associated with progression to ESRD [22]. Our observation of a high ESRD incidence rates in this population with relatively preserved kidney function at enrollment, highlights opportunities to identify and better characterize additional ESRD risk factors, including modifiers of the effect of APOL1.

Our study confirmed roles for diabetes and hypertension in kidney disease as observed in other large cohort studies such as the Framingham Heart/Offspring Study [23], Multiple Risk Factor Intervention Trial (MRFIT) [24], and Chronic Renal Insufficiency Cohort (CRIC) [25]. Strengths of our study include the availability of data on both baseline eGFR in addition to ESRD incidence. Furthermore, our study population included sizable numbers of blacks and women. The CRIC study included a significant number of blacks (42%), had a high prevalence of baseline cardiovascular risk factors (48% with diabetes and 86% with hypertension) and well-characterized baseline kidney function, and had a high ESRD incidence rate. By design, CRIC included a significant number (~ 20%) of participants who had significant kidney function impairment (eGFR < 30 ml/min/1.73m^2^) at baseline. In contrast, we observed a high incidence of ESRD in our study despite the large number of participants with a relatively preserved eGFR (70% had an eGFR > 90 at enrollment), perhaps signifying rapid progression over a 5–6 year period. There remains a need to characterize patterns of progression to ESRD and risk factors that may impact ESRD progression across a wide eGFR range in high risk groups.

For most levels of baseline eGFR < 100 ml/min/1.73m^2^ blacks had a higher ESRD risk than whites. While the CRIC study demonstrated an increased ESRD risks of blacks compared to whites (HR 1.55, 95% CI 1.29–1.86) after adjusting for baseline eGFR, we demonstrate that the difference in ESRD risk may diminish at an eGFR of less than 40. These data may indicate a need to target blacks for CKD screening, treatment, and therapeutic trials earlier in their disease course, when yet to be elucidated risk factors may play a significant role in progression. As CKD advances, common mechanisms of progression including hyperfiltration injury, elevated glomerular pressure, and fibrosis, may predominate in determining the path to ESRD. Most current clinical ESRD risk calculators do not include race as a variable. Future studies should examine whether race is helpful when estimating ESRD risk based on baseline eGFR level and time horizon. The general benefit of these approaches requires further evaluation [26, 27].

A well- known risk factor for CKD progression to ESRD is proteinuria [28]. Different levels of proteinuria have been associated with racial differences of CKD progression [29] and this may hypothetically account for some of the racial disparities in ESRD risk. However, the role of proteinuria in the disparity of ESRD incidence among blacks remains ill-defined and the unavailability of baseline urine protein levels precluded assessment of this risk factor in the SCCS. Additional limitations of our study warrant consideration. These include the self-reported nature of the baseline questionnaire, the lack of time-dependent covariates and the lack of follow-up creatinine measurements, which precluded calculation of eGFR slopes over time. Finally, residual confounding arising from inadequate control of diabetes or hypertension severity cannot be excluded. Only participants who enrolled in 2004 or later and responded affirmatively to the question of whether a doctor ever told them they have high blood pressure were asked additional follow-up questions on the use of prescription medications for blood pressure control; thus restricting our analyses to those who enrolled after 2004 yielded an insufficient sample size.

We have previously shown a survival advantage among blacks compared to whites in the SCCS, both overall [30] and among those with diabetes [31], but in sensitivity analyses taking into account competing risks, higher rates of ESRD in black males and black females do not appear to be the result of different rates of death between black and white subjects.

## Conclusions

In conclusion, we describe a cohort of blacks and whites with a high prevalence of baseline risk factors who, despite relatively high levels of baseline renal function, progress to ESRD. Furthermore, our study provides evidence that additional unknown risk factors account for the racial disparity of increased ESRD incidence in blacks, and demonstrates the need for early interventions that are not solely based on kidney function assessment in at-risk populations.

## Additional files

Additional file 1: **Table S1.** Baseline characteristics of ESRD cases and subcohort members according to category of eGFR (ml/min/1.73m^2)^, SCCS 2002–2009). (DOCX 30 kb)

Additional file 2: **Table S2.** Sensitivity Analysis: Cause-Specific Model Coefficients. (DOCX 21 kb)

Additional file 3: **Table S3.** Partial effect plot (Fig. 3) slopes. (DOCX 21 kb)

Additional file 4: **Figure S1.** 10-year probabilities for each race and gender combination for ESRD and death. (DOCX 119 kb)

## Data Availability

The datasets used and/or analysed during the current study are available from the corresponding author on reasonable request.

## Abbreviations

APOL1: Apoliproprotein L1
BMI: Body mass index
CI: Confidence interval
CKD: Chronic kidney disease
CRIC: Chronic Renal Insufficiency Cohort
eGFR: Estimated glomerular filtration rate
ESRD: End stage renal disease risk
HR: Hazard ratio
MRFIT: Multiple Risk Factor Intervention Trial
SCCS: Southern Community Cohort Study
SES: Socioeconomic status
USRDS: US Renal Data System

## References

[1] Go AS, Chertow GM, Fan D, McCulloch CE, Hsu CY (2004). Chronic kidney disease and the risks of death, cardiovascular events, and hospitalization. N Engl J Med.

[2] Neuen BL, Chadban SJ, Demaio AR, Johnson DW, Perkovic V (2017). Chronic kidney disease and the global NCDs agenda. BMJ Glob Health.

[3] Saran R, Robinson B, Abbott KC, Agodoa LY, Albertus P, Ayanian J (2017). US renal data system 2016 annual data report: epidemiology of kidney disease in the United States. Am J Kidney Dis.

[4] Albertus P, Morgenstern H, Robinson B, Saran R (2016). Risk of ESRD in the United States. Am J Kidney Dis.

[5] Bash LD, Astor BC, Coresh J (2010). Risk of incident ESRD: a comprehensive look at cardiovascular risk factors and 17 years of follow-up in the atherosclerosis risk in communities (ARIC) study. Am J Kidney Dis.

[6] Choi AI, Rodriguez RA, Bacchetti P, Bertenthal D, Hernandez GT, O'Hare AM (2009). White/black racial differences in risk of end-stage renal disease and death. Am J Med.

[7] McClellan WM, Warnock DG, Judd S, Muntner P, Kewalramani R, Cushman M (2011). Albuminuria and racial disparities in the risk for ESRD. J Am Soc Nephrol.

[8] Xue JL, Eggers PW, Agodoa LY, Foley RN, Collins AJ (2007). Longitudinal study of racial and ethnic differences in developing end-stage renal disease among aged medicare beneficiaries. J Am Soc Nephrol.

[9] Lipworth L, Mumma MT, Cavanaugh KL, Edwards TL, Ikizler TA, Tarone RE (2012). Incidence and predictors of end stage renal disease among low-income blacks and whites. PLoS One.

[10] Hargreaves MKAC, Blot WJ, Satcher DPR (2006). Community health centers: their role in the treatment of minorities and in health disparities research. Multicultural medicine and health disparities.

[11] Signorello LB, Hargreaves MK, Blot WJ (2010). The southern community cohort study: investigating health disparities. J Health Care Poor Underserved.

[12] Signorello LB, Hargreaves MK, Steinwandel MD, Zheng W, Cai Q, Schlundt DG (2005). Southern community cohort study: establishing a cohort to investigate health disparities. J Natl Med Assoc.

[13] Malhotra R, Cavanaugh KL, Blot WJ, Ikizler TA, Lipworth L, Kabagambe EK (2016). Higher protein intake is associated with increased risk for incident end-stage renal disease among blacks with diabetes in the southern community cohort study. Nutr Metab Cardiovasc Dis.

[14] Levey AS, Stevens LA, Schmid CH, Zhang YL, Castro AF, Feldman HI (2009). A new equation to estimate glomerular filtration rate. Ann Intern Med.

[15] Robinson-Cohen C, Zelnick LR, Hoofnagle AN, Lutsey PL, Burke G, Michos ED (2017). Associations of vitamin D-binding globulin and bioavailable vitamin D concentrations with coronary heart disease events: the multi-ethnic study of atherosclerosis (MESA). J Clin Endocrinol Metab.

[16] Therneau TM, Li H (1999). Computing the cox model for case cohort designs. Lifetime Data Anal.

[17] Sharp SJ, Poulaliou M, Thompson SG, White IR, Wood AM (2014). A review of published analyses of case-cohort studies and recommendations for future reporting. PLoS One.

[18] Romagnani P, Remuzzi G, Glassock R, Levin A, Jager KJ, Tonelli M (2017). Chronic kidney disease. Nat Rev Dis Primers.

[19] Estrella MM, Parekh RS (2017). The expanding role of APOL1 risk in chronic kidney disease and cardiovascular disease. Semin Nephrol.

[20] Genovese G, Friedman DJ, Ross MD, Lecordier L, Uzureau P, Freedman BI (2010). Association of trypanolytic ApoL1 variants with kidney disease in African Americans. Science..

[21] Heymann J, Winkler CA, Hoek M, Susztak K, Kopp JB (2017). Therapeutics for APOL1 nephropathies: putting out the fire in the podocyte. Nephrol Dial Transplant.

[22] Young BA, Fullerton SM, Wilson JG, Cavanaugh K, Blacksher E, Spigner C (2017). Clinical genetic testing for APOL1: are we there yet?. Semin Nephrol.

[23] Weiner DE, Tighiouart H, Amin MG, Stark PC, MacLeod B, Griffith JL (2004). Chronic kidney disease as a risk factor for cardiovascular disease and all-cause mortality: a pooled analysis of community-based studies. J Am Soc Nephrol.

[24] Brancati FL, Whelton PK, Randall BL, Neaton JD, Stamler J, Klag MJ (1997). Risk of end-stage renal disease in diabetes mellitus: a prospective cohort study of men screened for MRFIT. Multiple risk factor intervention trial. JAMA..

[25] Yang W, Xie D, Anderson AH, Joffe MM, Greene T, Teal V (2014). Association of kidney disease outcomes with risk factors for CKD: findings from the chronic renal insufficiency cohort (CRIC) study. Am J Kidney Dis.

[26] Grams ME, Sang Y, Ballew SH, Carrero JJ, Djurdjev O, Heerspink HJL (2018). Predicting timing of clinical outcomes in patients with chronic kidney disease and severely decreased glomerular filtration rate. Kidney Int.

[27] Eckardt KU, Bansal N, Coresh J, Evans M, Grams ME, Herzog CA (2018). Improving the prognosis of patients with severely decreased glomerular filtration rate (CKD G4+): conclusions from a kidney disease: improving global outcomes (KDIGO) controversies conference. Kidney Int.

[28] Iseki K, Ikemiya Y, Iseki C, Takishita S (2003). Proteinuria and the risk of developing end-stage renal disease. Kidney Int.

[29] Fischer MJ, Hsu JY, Lora CM, Ricardo AC, Anderson AH, Bazzano L (2016). CKD progression and mortality among Hispanics and non-Hispanics. J Am Soc Nephrol.

[30] Signorello LB, Cohen SS, Williams DR, Munro HM, Hargreaves MK, Blot WJ (2014). Socioeconomic status, race, and mortality: a prospective cohort study. Am J Public Health.

[31] Conway BN, May ME, Fischl A, Frisbee J, Han X, Blot WJ (2015). Cause-specific mortality by race in low-income black and White people with type 2 diabetes. Diabet Med.

