# Supplementary material for: Racial disparities in end-stage renal disease in a high-risk population: the Southern Community Cohort Study
Source: BMC Nephrol. 2019 Aug 7;20:308. doi: 10.1186/s12882-019-1502-z (PMC6686512; doi:10.1186/s12882-019-1502-z)
Supplement: Supplementary file 1 — Table S1. Baseline characteristics of ESRD cases and subcohort members according to category of eGFR (ml/min/1.73m2), SCCS 2002–2009). (DOCX 30 kb) [file 12882_2019_1502_MOESM1_ESM.docx]

**Table S1**. Baseline characteristics of ESRD cases and subcohort members according to category of eGFR (ml/min/1.73m^2)^, SCCS 2002-2009).

| **Variable** | **eGFR ≤30** | | **eGFR 31-60** | | **eGFR 61-90** | | **eGFR ≥90** | |
| --- | --- | --- | --- | --- | --- | --- | --- | --- |
|  | **S** | **ESRD** | **S** | **ESRD** | **S** | **ESRD** | **S** | **ESRD** |
| Age (median,  25^th^, 75^th^ percentile) | 59  (52, 68) | 55  (50, 61) | 55  (48, 63) | 52  (48, 57) | 56  (51, 60) | 56  (50, 60) | 48  (44, 52) | 50  (46, 58) |
| Age categories (%)  40-49  50-59  60-69  70-79 | 19.4  32.4  27.6  20.5 | 29.2  41.8  21  8.1 | 32.2  37.1  23  7.7 | 32.9  56.8  8.5  1.8 | 24.7  52.4  13.3  9.7 | 27.6  51.1  18.3  2.9 | 63.5  28.9  6.9  0.6 | 52.5  33.6  12.8  1.1 |
| Race + Sex categories (%)  Black female  Black male  White female  White male | 37.8  24.6  29.8  7.8 | 41.6  42.9  11.6  4 | 36.7  25.9  24.2  13.3 | 48.8  45.6  3.9  1.8 | 47.8  31.9  13.3  7 | 55.6  32  9.1  3.3 | 45.0  41.4  8.3  5.3 | 33.7  49.5  10.5  6.3 |
| Marital status (%)  Married  Separated  Single  Widowed | 35.1  28.7  14.1  22.1 | 32.9  35.7  15.3  16.1 | 34.7  33.3  17.1  14.9 | 39.3  31.3  19.4  10 | 25.9  28.3  31.2  14.6 | 27.5  31.1  29.2  12.1 | 31.1  32.9  29.3  6.6 | 31  28.5  30  10.5 |
| Education < 12th grade, (%) | 36 | 43.9 | 36 | 49.2 | 47 | 43 | 31.7 | 29.3 |
| Income ≤$15,000 (%) | 61.3 | 67.7 | 62.3 | 59.6 | 65.1 | 57.7 | 65.6 | 77.5 |
| BMI (Median, IQR) | 31  (26, 34) | 32  (27, 37) | 30  (25, 35) | 33  (27, 42) | 31  (26, 36) | 32  (26, 41) | 31  (26, 37) | 29  (24, 35) |
| BMI categories  Underweight (<18.5)  Normal (18.5-24.9)  Overweight (25-29.9)  Obese (30+) | 0  18.2  28.5  53.4 | 0.6  15.4  21.9  62 | 1.3  20.2  29.6  48.9 | 1.1  7.6  30  61.3 | 0  17.5  32  50.5 | 0  19.4  25.9  54.7 | 0.6  16.5  28.7  54.3 | 0.3  27.2  32.1  40.4 |
| Smoking status (%)  Current  Former  Never | 31.7  28.8  39.4 | 31.6  28.4  40 | 36.8  25.9  37.4 | 24.9  28.3  46.7 | 27.2  34.7  38.1 | 18  23.2  58.8 | 41.9  16.2  41.9 | 56.3  17.1  26.7 |
| Hypertension (%) | 83.2 | 90.2 | 63.7 | 87.3 | 99.6 | 95.8 | 77.2 | 73.8 |
| Diabetes (%) | 34 | 61.8 | 22.2 | 66.7 | 66.2 | 69.1 | 78.4 | 76.5 |
| Stroke, TIA (%) | 11.8 | 15.5 | 7.4 | 8.3 | 7 | 17.1 | 9.6 | 10.4 |

Abbreviations: BMI, body mass index; eGFR, estimated glomerular filtration rate; ESRD, end-stage renal disease; MI, myocardial infarction; S, subcohort; SCCS, Southern Community Cohort Study; TIA, transient ischemic attack
