# Supplementary material for: Racial disparities in end-stage renal disease in a high-risk population: the Southern Community Cohort Study
Source: BMC Nephrol. 2019 Aug 7;20:308. doi: 10.1186/s12882-019-1502-z (PMC6686512; doi:10.1186/s12882-019-1502-z)
Supplement: Supplementary file 2 — Table S2. Sensitivity Analysis: Cause-Specific Model Coefficients. (DOCX 21 kb) [file 12882_2019_1502_MOESM2_ESM.docx]

**Table S2**. Sensitivity Analysis: Cause-Specific Model Coefficients.

| **Variable** | **Enrollment 🡪 ESRD** | **Enrollment 🡪 Death** |
| --- | --- | --- |
| Black (Ref: White) | 0.49 | 0.1 |
| Female (Ref: Male) | -0.61 | -0.49 |
| Diabetes (Ref: No) | 1.6 | 0.17 |
| Hypertension (Ref: No) | 0.43 | -0.05 |
