# Supplementary material for: Racial disparities in end-stage renal disease in a high-risk population: the Southern Community Cohort Study
Source: BMC Nephrol. 2019 Aug 7;20:308. doi: 10.1186/s12882-019-1502-z (PMC6686512; doi:10.1186/s12882-019-1502-z)
Supplement: Supplementary file 3 — Table S3. Partial effect plot (Fig. 3) slopes. (DOCX 21 kb) [file 12882_2019_1502_MOESM3_ESM.docx]

**Table S3**. Partial effect plot (Fig. 3) slopes

| **eGFR** | **Slope: Blacks** | **Slope: Whites** |
| --- | --- | --- |
| 15-30 | -0.71 | -1.13 |
| 30-45 | -0.75 | -1.19 |
| 45-60 | -0.75 | -1.19 |
| 60-75 | -0.68 | -1.02 |
| 75-90 | -0.58 | -0.71 |
| 90-105 | -0.37 | -0.13 |
| 105-120 | -0.22 | 0.34 |
| 120-135 | -0.18 | 0.43 |

eGFR in ml/min/1.73m^2^
