# Supplementary figures and images for: Racial disparities in end-stage renal disease in a high-risk population: the Southern Community Cohort Study
Source: BMC Nephrol. 2019 Aug 7;20:308. doi: 10.1186/s12882-019-1502-z (PMC6686512; doi:10.1186/s12882-019-1502-z)

**Figure S1.** 10-year probabilities for each race and gender combination for ESRD and death


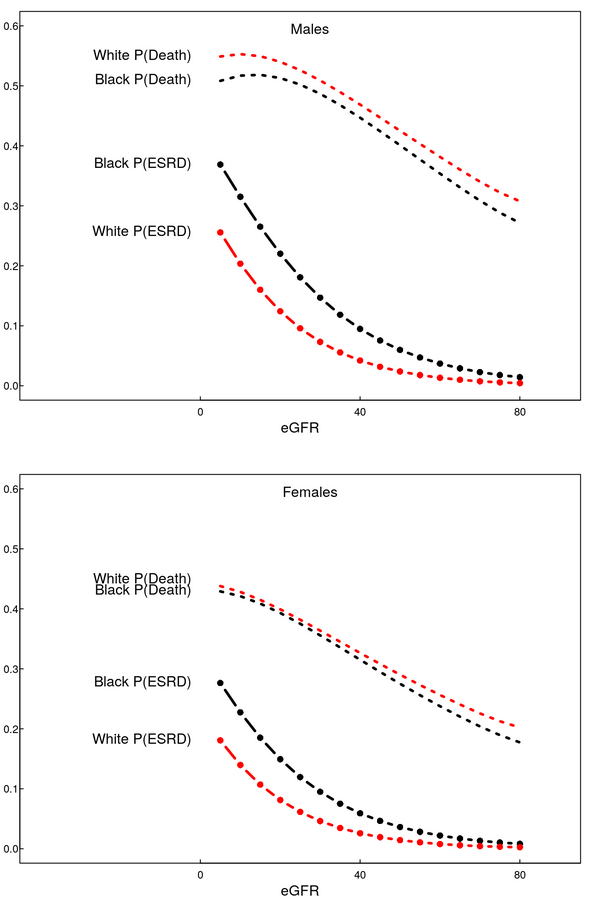

Supplement: Supplementary file 4 — Figure S1. 10-year probabilities for each race and gender combination for ESRD and death. (DOCX 119 kb) [file 12882_2019_1502_MOESM4_ESM.docx]
